# Vertebrate Dissimilarity Due to Turnover and Richness Differences in a Highly Beta-Diverse Region: The Role of Spatial Grain Size, Dispersal Ability and Distance

**DOI:** 10.1371/journal.pone.0082905

**Published:** 2013-12-04

**Authors:** Jaime M. Calderón-Patrón, Claudia E. Moreno, Rubén Pineda-López, Gerardo Sánchez-Rojas, Iriana Zuria

**Affiliations:** 1 Centro de Investigaciones Biológicas, Instituto de Ciencias Básicas e Ingeniería, Universidad Autónoma del Estado de Hidalgo, Pachuca, Hidalgo, México; 2 Facultad de Ciencias Naturales, Universidad Autónoma de Querétaro, Juriquilla, Querétaro, México; University of Waikato (National Institute of Water and Atmospheric Research), New Zealand

## Abstract

We explore the influence of spatial grain size, dispersal ability, and geographic distance on the patterns of species dissimilarity of terrestrial vertebrates, separating the dissimilarity explained by species replacement (turnover) from that resulting from richness differences. With data for 905 species of terrestrial vertebrates distributed in the Isthmus of Tehuantepec, classified into five groups according to their taxonomy and dispersal ability, we calculated total dissimilarity and its additive partitioning as two components: dissimilarity derived from turnover and dissimilarity derived from richness differences. These indices were compared using fine (10 x 10 km), intermediate (20 x 20 km) and coarse (40 x 40 km) grain grids, and were tested for any correlations with geographic distance. The results showed that total dissimilarity is high for the terrestrial vertebrates in this region. Total dissimilarity, and dissimilarity due to turnover are correlated with geographic distance, and the patterns are clearer when the grain is fine, which is consistent with the distance-decay pattern of similarity. For all terrestrial vertebrates tested on the Isthmus of Tehuantepec both the dissimilarity derived from turnover and the dissimilarity resulting from richness differences make important contributions to total dissimilarity, and dispersal ability does not seem to influence the dissimilarity patterns. These findings support the idea that conservation efforts in this region require a system of interconnected protected areas that embrace the environmental, climatic and biogeographic heterogeneity of the area.

## Introduction

The term beta diversity describes changes or variations in species composition, and is quite relevant in ecology and biogeography because it allows us to test hypotheses about the processes that drive species distribution, thus it is a key concept for biodiversity conservation and ecosystem management [1]. Beta diversity is influenced by three main factors: the organism’s dispersal ability, spatial scale—including variations in the grain and/or the extent of the study according to Barton et al. [2]-, the biogeographic history of the species, and niche limitations. When dispersal ability is limited, beta diversity tends to be high [3]. For example, among terrestrial vertebrates the lowest beta diversity occurs in birds, which have the highest vagility. Mammals and reptiles have intermediate values of beta diversity, and amphibians have the highest values, due to their limited dispersal ability [4-6]. Regarding scale, it has been shown that large areas studied using a coarse grain have lower turnover than small areas do [5,7,8]. This may occur because the number of shared species increases as grain size increases, because large areas have more species, or because the degree of aggregation in the distribution of species also decreases [9]. Biogeographic history also plays a crucial role in the patterns of beta diversity. For example, latitudinal variation in beta diversity may result from temperature fluctuations during glaciation events, and the corresponding processes of extinction and colonization [6,10,11].

 Mexico is one of the most biodiverse countries in the world. However, unlike other countries, its high biological diversity does not depend on high values of local species richness, but rather is determined by exceptionally high beta diversity [12,13]. When the spatial patterns of biodiversity in the country are analyzed, amphibians and reptiles have the highest values of beta diversity, followed by mammals, while birds have the lowest values of beta diversity among terrestrial vertebrates [4]. These results are clearly related to dispersal ability, and to the mean size of the species distribution range: as the distribution range decreases, beta diversity increases [4]. Also, if mammals are divided according to their differences in vagility, nonflying mammals have higher values of beta diversity than bats, which in general have higher dispersal abilities and larger distribution ranges [13].

**Figure 1:**
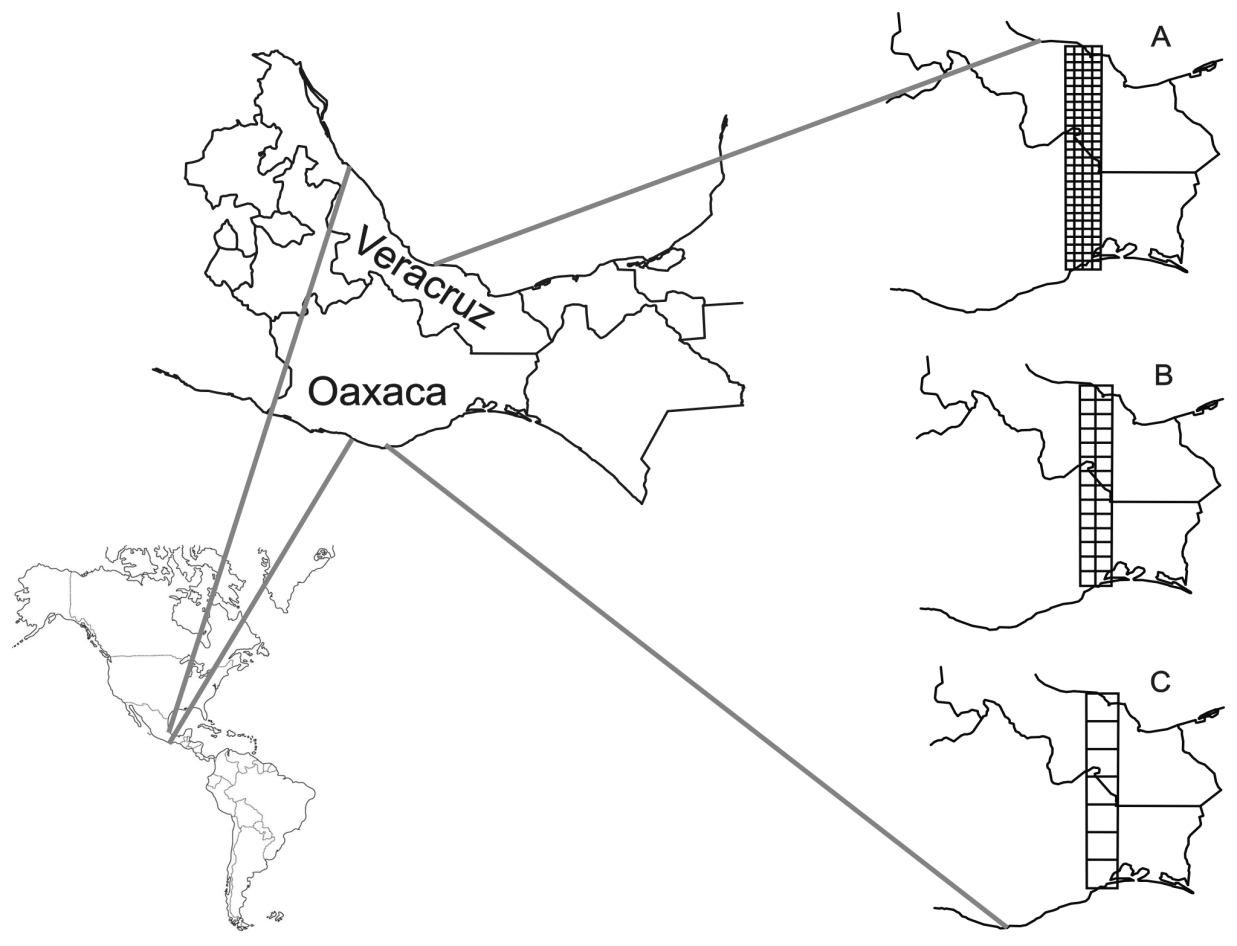
Study area on the Isthmus of Tehuantepec, Mexico. We show the grids used for the analyses at three grain sizes. A: fine grain, 10 x 10 km cells, B: intermediate grain, 20 x 20 km cells, and C: coarse grain, 40 x 40 km cells.

 In Mexico, values of biodiversity are high on the Isthmus of Tehuantepec in the southern states of Chiapas, Oaxaca and Veracruz (Figure 1). The isthmus is a narrow region of lowlands with a minimum width of 200 km, and a maximum elevation of 250 m a.s.l. at the central part, which forms a 40-km-wide plain that separates the highlands of southern Mexico from the highlands of Chiapas and Guatemala, and the highlands of Central America. This region functions as a biological corridor for species from the Gulf of Mexico and the Pacific plains [14-16]. The region is recognized as an important biogeographic node where historic events have shaped the transition between the Nearctic and the Neotropical regions [17,18]. According to the geological evidence, there was an important tectonic event in the Pliocene at the Isthmus of Tehuantepec, when the highland corridors that occurred in the Miocene were destroyed by extreme tectonic activity related to the subduction of the Cocos Plate [16,19,20]. During this period, snakes of the genera *Atropoides*, *Botriechis*, and *Cerrophidium* diverged in the region [17], as did some species of birds [18] and rodents [20,21]. Thus, this biogeographic node has played a complex role in modulating the historic gene flow [17]. This region currently has one of the highest concentrations of vertebrate species in Mexico [4]. For example, only for the portion of the Isthmus of Tehuantepec that corresponds to the state of Oaxaca more than 150 species of amphibians and reptiles have been recorded [22], along with 362 species of birds [23] and 102 mammals [24]. Moreover, given its high heterogeneity, as a region the Isthmus of Tehuantepec has the second highest values of mammal beta diversity in the country, after the central part of Mexico [24]. This high beta diversity in the area has also been described for plants and for the different groups of terrestrial vertebrates [4].

 In the last few years the study of beta diversity has benefited from different concepts and new methods of analysis 1 of the more practically applicable contributions is the partition of total dissimilarity into two components: the dissimilarity derived from species turnover and that derived from richness differences [10,25-28]. This partitioning has been recently used, following different methodological approaches, by Leprieur et al. [29], Dobrovolski et al. [6], Baselga et al. [11], and Boeiro et al. [30], who examined the contribution of turnover and richness differences for different biological groups to evaluate the influence of various processes on the patterns of beta diversity.

The aim of this study is to analyze the patterns of dissimilarity for the terrestrial vertebrates of the Isthmus of Tehuantepec, separating dissimilarity caused by turnover from that caused by richness differences, in order to explore the effect of spatial grain size, vertebrate dispersal ability, and the geographic distance between observational units. We perform separate analyses for five biological groups, at the taxonomic level of Class, and use the different dispersal abilities of these groups to examine their effect on the results [13,31]: 1) amphibians, 2) reptiles, 3) birds, 4) nonflying mammals, and 5) bats. 

Based on the idea that beta diversity has two components, the turnover and the richness differences, we expect to find the following trends: a) in general, total dissimilarity will be high for all groups, and the contribution of species turnover will be stronger given that the isthmus is one of the areas that has the most species with a restricted distribution range in Mexico; b) spatial grain will be inversely related to dissimilarity: when the grain is small we expect to detect higher values of dissimilarity, while at intermediate and coarse grains dissimilarity will decrease; c) dissimilarity will be inversely related to the dispersal ability of vertebrates, thus, amphibians will have the highest values of dissimilarity, and in descending order they will be followed by reptiles, nonflying mammals, bats, and birds; and d) given the distance-decay pattern of similarity, we will find significant correlations between dissimilarity and geographic distance, and those relationships will be stronger at finer grains.

## Materials and Methods

### Ethics Statement

Field samplings were done on private lands, with the corresponding permission of owners. Field sampling was authorized by the Secretaría del Medio Ambiente y Recursos Naturales (Mexican Council for the Environment and Natural Resources), which legislates scientific field samplings in Mexico, through permission 06108/09, according to the document SEMARNAT-08-049-A. We did not perform any other activities that required specific permissions, and the field sampling did not involve endangered or protected species. We did not sacrifice any organism for this study, because all of the individuals were released in the same location of capture. For field sampling in Mexico, the approval by an Institutional Animal Care and Use Committee (IACUC) or equivalent animal ethics committee is not required.

### Field Sampling

Data were gathered from direct field sampling and from databases and literature review (see next section). Field work was carried out from July to September 2009 in 14 sampling sites along a transect of ca. 130 km, covering five vegetation types. For sampling anurans and reptiles, we carried out a direct search during day and night periods (from 9:00 to 13:00, 16:00 to 18:00, and 20:00 to 01:00). We captured individuals by hand or using herpetological hooks while animals were on the floor, below rocks, in caves, or ponds. 

For birds, we did bird counts and captured individuals with mist nets. Bird counts were carried out from 17:00 to 19:00 h. At each sampling site a ca. 300 m transect was walked slowly for 20 min recording all birds seen. We captured birds using seven mist nets (12 x 3 m) at each sampling site from 6:30 to 12:30 h, in two consecutive days. 

We used Sherman livetraps for capturing terrestrial small mammals, camera traps and sign surveys (tracks and scats) for medium sized mammals, and mist nets for bats. At each sampling site, 50 Sherman traps baited with oats and vanilla extract were set on a 500 m transect; the distance between traps was 10 m. Traps were active for two consecutive nights. We located 22 camera traps along trails that remained active for five months,; the cameras were checked every 20 days. Tracks and scats were recorded over non-restricted day and night walks at each sampling site. Finally, for bats we used five 12 x 3 m mist nets at each sampling site, that remained open over two consecutive nights from 19:00 to 00:00. 

### Biological Data and Study Area Delimitation

We compiled a database of vertebrate species for the Isthmus of Tehuantepec using georeferenced data for the states of Oaxaca and Veracruz (Fig. 1) from different sources, including literature and digital databases with information from scientific collections in Mexico and abroad: GBIF (Global Biodiversity Information Facility, http://www.gbif.org), UNIBIO (*Unidad de Informática para la Biodiversidad* of the UNAM’s Institute of Biology, http://unibio.unam.mx), and records provided by CONABIO (*Comisión Nacional para el Uso y Conocimiento de la Biodiversidad*: projects A14, A26, A27, AA3, B2, CC2, CE6, DC5, DC6, E18, G15, H245, J121, J123, L47, P130, P132, P60, UAZ, R246, S137, T9, U14, V9, W36). We then reviewed the current taxonomic nomenclature and the distribution of each species in specialized literature [23,32-37], the webpage of the American Ornithologist’s Union (http://www.aou.org) and that of the Amphibian Species of the World (http://research.amnh.org). Finally, we discarded all duplicate or triplicate records gathered from our different information sources, and kept only one record. Thus, our analyses do not include duplicate records.

In order to delimit the study area, we set up a grid with cells measuring 0.083 x 0.083 degrees. This grid size was used to clearly observe the cells in which most of the records are concentrated, and because this grain size has also been used in diversity analyses done at the national level [4,38]. For each cell we calculated the density of records, using the neighborhood method, excluding those cells with a low concentration of records from the analysis. The results showed that the areas with the highest density of records are located along the *Panamericana Federal Transítsmica* #185 highway. After delimiting the study area (i.e. fixed extent), we proceeded to divide the surface using grids of varying grain size, following one of the conceptual approaches recently revised by Barton et al. [2]. We used grids with three grain sizes: 10 x 10 km (122 cells), 20 x 20 km (28 cells) and 40 x 40 km (seven cells), which we refer to as fine, intermediate, and coarse grain hereafter (Figure 1).

For each cell we counted the number of species and the total number of records of each biological group. Cell species richness varied from 0 to 108 (the maximum number was for reptiles in the Los Tuxtlas region). Cells with no records were not included in the analysis. With this information we built matrices with species presence-absence information, and with the number of records for each one of the five vertebrate groups.

Finally, we identified the cells for which species inventories were adequately complete, using the total number of records of all the species as a measure of sampling effort [38]. For each biological group and grain size, inventory completeness was measured as the sample coverage percentage, which in our context is the proportion of the total number of records in a cell that belong to the species represented in our database [39]. To avoid under-sampling biases, only those cells with inventory completeness equal to or greater than 75% were used in the dissimilarity analysis.

Inventory completeness at the fine grain level was greater than 75% in 23 cells for amphibians, in 21 cells for reptiles, 12 for birds, 24 for nonflying mammals, and 30 for bats. For the intermediate grain grid the number of cells with complete inventories was: 12 for amphibians, 13 for reptiles, eight for birds, 15 for nonflying mammals, and 14 for bats. For the coarse grain all seven cells were analyzed for reptiles, nonflying mammals and bats, but only six cells for amphibians and four for birds reached the requested sample coverage (Figure S1). The final database includes 67 species of amphibians, 203 reptiles, 459 birds, 89 nonflying mammals, and 87 bats, for a total of 905 terrestrial vertebrate species (Checklist S1).

### Data Analysis

Total dissimilarity was calculated with the Jaccard index (β_cc_ sensu [26,28], β_jac_ sensu [25]), and it was broken into two additive components: the dissimilarity derived from species turnover or replacement (β_-3_) and the dissimilarity derived from richness differences (β_rich_), following the framework proposed by J. C. Carvalho [26,28]. The sum of the values derived from turnover and richness differences must be equal to total dissimilarity, which ranges from 0 to 1. These indices were calculated using R [40] with a specific script [28].

We assessed differences in total dissimilarity (β_cc_), turnover dissimilarity (β_-3_) and richness differences dissimilarity (β_rich_) among the five biological groups for each grain size by means of Kruskal-Wallis tests. When statistical differences were found, we performed Bonferroni corrected pairwise comparisons.

To test whether dissimilarity changes with spatial grain size, we compared the mean values of total dissimilarity (β_cc_), turnover dissimilarity (β_-3_) and richness differences dissimilarity (β_rich_), among the three grain sizes using one way ANOVA tests, with Tukey tests for pairwise multiple comparisons in case of significant differences. As these values are means, they passed the normality test, so there was no need to transform the data. We calculated the geographic distance between cells using the centroid method, locating the central point of each cell and measuring its distance to the other central points, for the three grain sizes. Finally, for each biological group and each spatial grain size, we assessed the relationships between geographic distance and dissimilarity (β_cc_, β_-3_ and β_rich_) using Mantel correlations. The ANOVA and Mantel test were run in the Past program [41].

## Results

**Figure 2:**
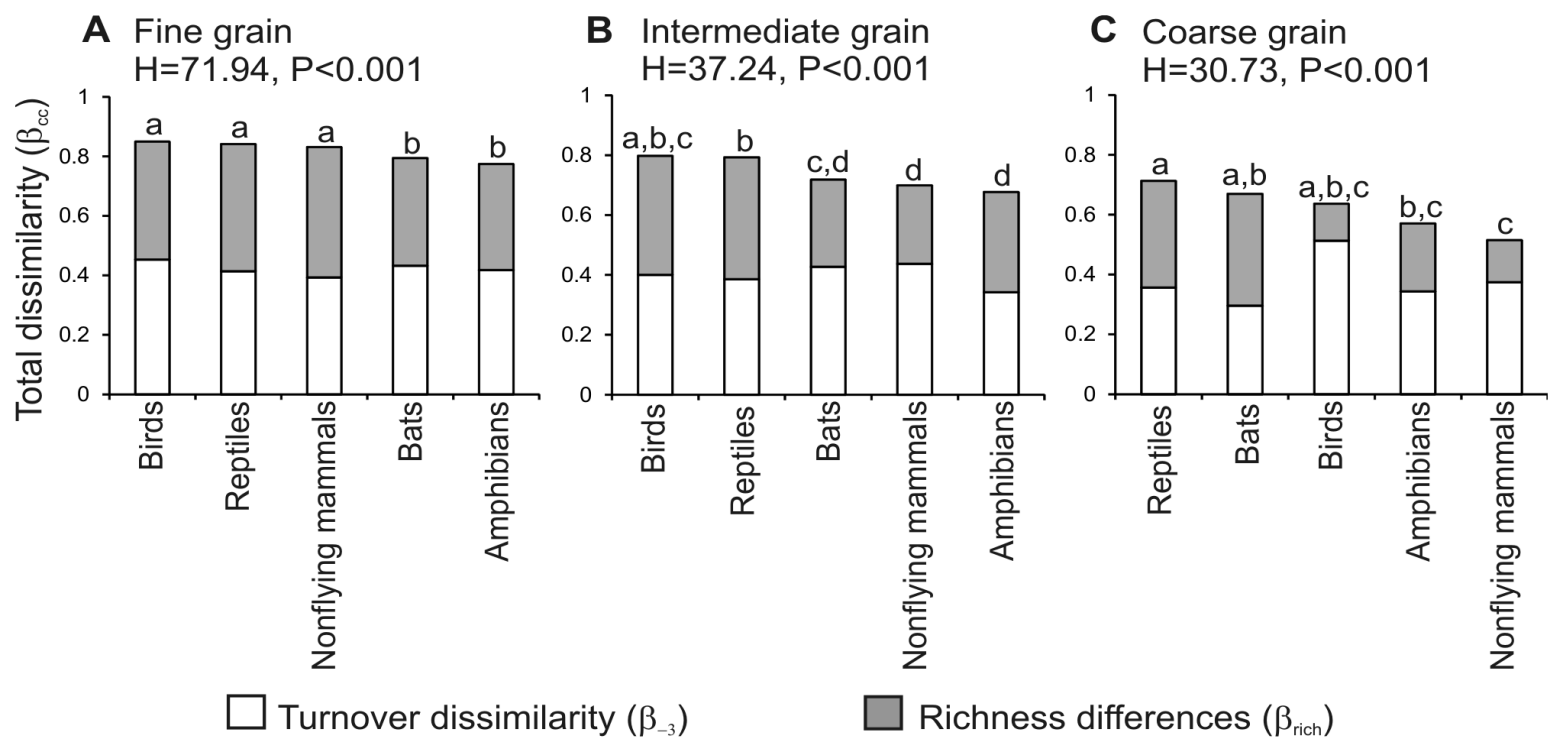
Relative contribution of species turnover and richness differences to the total dissimilarity. The graph shows this contribution for different groups of terrestrial vertebrates on the Isthmus of Tehuantepec, using three grain sizes. Kruskal-Wallis results for total dissimilarity are included, and equal low case letters indicate no difference in pairwise comparisons. Tests results for species turnover and richness differences are given in the text.

Our results confirm that for terrestrial vertebrates, the Isthmus of Tehuantepec is a highly beta-diverse region, and we found that total dissimilarity was due to both species turnover and richness differences (Figure 2).

For the finest grain, mean total dissimilarity (β_cc_) was 82% for the five groups of vertebrates, with significant differences among groups (Figure 2a): birds, reptiles and nonflying mammals had higher total dissimilarity than bats and amphibians. From this total dissimilarity, the dissimilarity due to species turnover (β_-3_) ranged from 0.39 in nonflying mammals to 0.45 in birds, but this contribution of turnover to total dissimilarity did not vary significantly among groups (H=7.71, P=0.10). Dissimilarity derived from richness differences (β_rich_) was different among the biological groups (H=23.56, P<0.01), it had the lowest value for bats (0.36) and the highest for nonflying mammals (0.44, Figure 2a). In this latter group we found the highest contribution of richness differences to total dissimilarity (52.68%).

For the intermediate grain, total dissimilarity also varied among groups (Figure 2b), the minimum value was 0.68 for amphibians, and reached 0.80 for birds (Figure 2b). At this grain size, the dissimilarity derived from species turnover was different among groups (H=10.63, P=0.03), it went from 0.34 in amphibians to 0.44 in nonflying mammals, and the only detectable difference in pairwise comparisons was between these two groups. The dissimilarity derived from richness differences again varied among groups (H=13, P=0.01), and it went from 0.26 in nonflying mammals to 0.41 in reptiles (Figure 2b). At this scale, turnover contributed the most (62.56%) to total dissimilarity in nonflying mammals, and richness differences were up to 52.36% of the total dissimilarity for reptiles.

For the coarse grain, total dissimilarity was different among groups, and ranged from 0.52 in nonflying mammals to 0.71 in reptiles (Figure 2c). Dissimilarity due to turnover did not vary statistically among the biological gropus (H=6.81, P=0.15), although it ranged from 0.30 in bats to 0.51in birds (representing 80.56% of total dissimilarity). Richness differences were different among groups (H=20.98, P=0.003), ranging from 0.12 in birds to 0.37 in bats (55.77 of total dissimilarity, Figure 2c). 

### Dissimilarity and Dispersal Ability

Contrary to our expectations, dispersal ability does not exert a clear influence on species dissimilarity. In fact, as shown in Figure 2, birds have the highest dissimilarities at fine and intermediate grains, even when this group is assumed to be the most vagile. In contrast, amphibians have very low values of dissimilarity for all three grain sizes, in spite of having the most limited dispersal ability. 

### Dissimilarity and Spatial Grain Size

**Figure 3:**
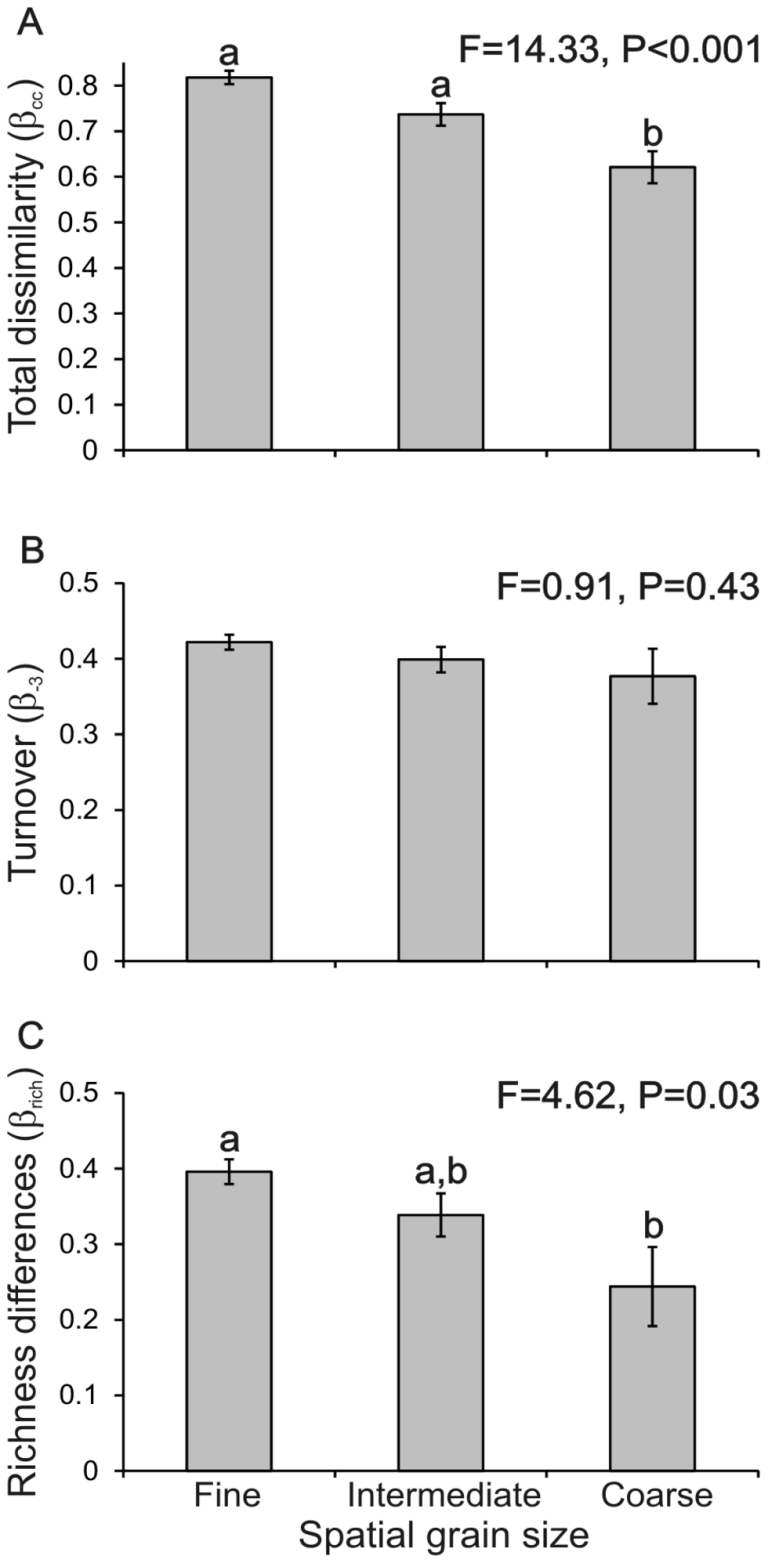
Components of dissimilarity at three spatial grain sizes. The figure shows mean total dissimilarity (A), dissimilarity derived from species turnover (B), and dissimilarity derived from richness differences (C) for terrestrial vertebrates on the Isthmus of Tehuantepec. Error bars are standard errors. ANOVA results are included, equal low case letters indicate no difference in pairwise comparisons.

According to our prediction, grain size is directly related to species dissimilarity, as shown in Figures 2 and 3. Using a fine grain we detect higher values of dissimilarity, while a coarse grain returns lower values of dissimilarity. Total dissimilarity (β_cc_) and dissimilarity derived from richness differences (β_rich_) changed significantly, decreasing as spatial grain size increased (Figure 3). In contrast, mean dissimilarity due to species turnover (β_-3_) was not different for the three grain sizes (Figure 3b). 

### Dissimilarity and Geographic Distance

Using the finest grain, total dissimilarity (β_cc_) is positively and significantly correlated with geographic distance for all groups of vertebrates, though correlation coefficients are low in some cases (Table 1). The correlation of β_cc_ is strongest for amphibians, and it was the only group for which geographic distance was significantly correlated with richness differences (β_rich_, Table 1).

**Table 1:** Mantel correlation coefficients between matrices of total dissimilarity (β_cc_), dissimilarity due to turnover (β_-3_) and dissimilarity due to richness differences (β_rich_), and the geographic distance between sites, for three spatial grain sizes.

	**Β_cc_**	**β_-3_**	**β_rich_**
**Fine grain: 10 x 10 km**			
Amphibians	0.6030 ***	0.1332 *	0.2430 *
Reptiles	0.4764 ***	0.3255 ***	-0.0642 ns
Birds	0.3928 ***	0.2958 ***	-0.1083 ns
Nonflying mammals	0.2698 ***	0.0767 ns	0.0567 ns
Bats	0.2358 ***	0.0955 *	0.0333 ns
**Intermediate grain: 20 x 20 km**			
Amphibians	0.6929 ***	0.3948 ***	0.1586 ns
Reptiles	0.3824 ***	0.4486 ***	-0.1925 ns
Birds	0.3560 *	0.1984 ns	0.0184 ns
Nonflying mammals	0.5471 ***	0.2743 **	0.0969 ns
Bats	0.2080 ns	0.1146 ns	0.0293 ns
**Coarse grain: 40 x 40 km**			
Amphibians	0.7260 **	0.1856 ns	0.2740 ns
Reptiles	0.3108 ns	0.3181 ns	-0.1356 ns
Birds	0.1288 ns	-0.5803 ns	0.9246 ns
Nonflying mammals	0.6421 *	0.5410 ***	-0.3252 ns
Bats	-0.0670 ns	0.1589 ns	-0.1633 ns

*P* values are *: *P*<0.05, **: *P*<0.01, ***: *P*<0.005, ns: not significant (*P*>0.05).

 Using the data from the intermediate grain, total dissimilarity and dissimilarity due to turnover were positively and significantly correlated with geographic distance for most of the groups, with the strongest correlation for the total dissimilarity of amphibians. However, none of the correlations with dissimilarity due to richness differences was significant (Table 1).

Finally, using the coarse grain grid total dissimilarity was positively and significantly correlated with geographic distance for amphibians and nonflying mammals (Table 1). For this latter group we also found a significant correlation between distance and dissimilarity derived from species turnover, but for none of the biological groups did we find a significant correlation between distance and richness differences (Table 1).

## Discussion

### The Isthmus of Tehuantepec: a Region with Notably High Beta Diversity

The results confirm our first prediction: beta diversity is very high in this region, with values above 0.77. And species dissimilarity is clearly derived from both species turnover and richness differences. This may be influenced by several factors. First, the biogeographic history of the isthmus had an effect when the highlands were considerably altered by tectonic activity, and became flatlands [16,19]. Also, given its location in tropical latitudes, the area did not experience drastic climate changes during glaciations, as those recorded in temperate regions. This favored the speciation of some vertebrates. In this biogeographic context, the current environmental conditions include a highly heterogeneous landscape, which shapes the distribution of the vegetation. Important climate factors in the region include environmental humidity and the flow of air masses from the coasts of the Gulf of Mexico and the Gulf of Tehuantepec, across the region in the absence of geographic barriers, as well as the strong north winds that cross the plains of the isthmus, resulting from gradients of atmospheric pressure [14]. Also, as a tropical region the study area has high productivity, and in areas with large energy availability (measured as annual potential evapotranspiration) the beta diversity of terrestrial vertebrates is higher [42]. All these factors contribute to climatic and environmental heterogeneity, resulting in a fine subdivision of the region into three physiographic subprovinces [15].

It is possible that speciation and the constant movement of vertebrates in the region hampered the assemblage of nested faunas to some extent, and thus the dissimilarity due to richness differences is not too high. This is quite different from the patterns reported for northern Europe and North America, where glaciation caused the phenomena of extinction and colonization, influencing the structure of current beetle and vertebrate faunas [6,10], but see 45, who also discusses the influence of non-climatic drivers in nestedness.

### The Highest Beta Diversity is Detected Using a Fine Spatial Grain

Our results corroborate the trend of increasing dissimilarity as grain size decreases. This is one of the most consistent of the patterns of beta diversity that have been reported for several groups, including insects and terrestrial vertebrates [5,8,24,43]. This is because as grain size increases, environmental differences between sampling units decrease, and more species are shared between the sample units. Moreover, bigger areas have more species and the degree of aggregation in species distribution is lower. Besides, there might be an influence of sampling biases: coarse grain may imply less undersampling, and therefore less beta diversity [44].

Also, with a coarse grain biogeographic history may explain much of the variation in diversity patterns and in the change in species composition [2,8,9]. In contrast, with a fine grain size, environmental heterogeneity is more important as a driver of changes in species composition at local scales [2,5,7,9,10,45].

### Dispersal Ability Does Not Determine the Dissimilarity for Vertebrates on the Isthmus of Tehuantepec

Contrary to the results of several terrestrial vertebrate studies that report higher beta diversity in organisms with low dispersal ability [4-6,8,43], in this study birds had high beta diversity (except for the dissimilarity due to species richness at the coarse grain size) while amphibians had low dissimilarity. Thus, dispersal ability does not seem to be a strong determinant of the beta diversity value of terrestrial vertebrates on the Isthmus of Tehuantepec. For birds, the isthmus is an obligatory route during their annual migration [18]. Of course, this has an impact on the high bird species richness in the area, and may be an important driver of the high beta diversity. To further explore this possibility, it would be interesting to perform separate analyses for different subgroups of birds, such as residents, migrants, and the assemblages that coexist during different seasons of the year. Our analyses were gross in the sense that we treated all bird species as a single group. However, we predict that if migratory birds were excluded, a more reliable pattern of beta diversity could have appeared. Thus, we suggest that further studies should focus on resident species, and assess the potentially different responses of functional groups. 

 For amphibians, the low total dissimilarity is probably related to the low species richness recorded in the majority of the cells, because rare species are occasional. Most of the cells include species with widespread distributions, and rare species are the key elements that may increase beta diversity. Amphibians are particularly difficult to survey (as compared to other vertebrates) because their detectability is biased by the experience of researchers and particular climatic conditions. Thus, a more thorough survey requires trained personal and extended sampling periods. Moreover, in our study area the records of species with restricted distributional ranges occur in the northern part of the isthmus, mainly in the region of Los Tuxtlas (see Figure S1). Thus, we need to improve amphibian inventories in the central and southern part of the Isthmus of Tehuantepec to determine whether low beta diversity is a generalized characteristic, or these results are due to sample biases.

For nonflying mammals and bats the differences in beta diversity may be related to the latitude of our study area. Latitude has a different effect on the beta diversity of North American mammals [45]: beta diversity of nonflying mammals decreases as latitude increases, while beta diversity of bats remains constant. Thus, at the latitude of the Isthmus of Tehuantepec beta diversity of bats is higher than that of nonflying mammals [46], as occurs with total dissimilarity at the intermediate and coarse grains in our study. Therefore, the effect of dispersal ability among flying and nonflying mammals may be obscured by the differential effects of latitude on beta diversity.

It is worth mentioning that the beta diversity studies on the dispersal ability of terrestrial vertebrates have been done on global, continental or national scales [4-6,8,43]. At these scales, changes in energy availability and in the environment are more evident. But for the regional scale of our work, energy availability does not appear to have an important effect because this is the narrowest region of the country and lacks geographic barriers. In spite of this, the relationship between dissimilarity and geographic distance may indicate some degree of influence by the organisms’ dispersal ability (discussed in the next section).

### Dissimilarity Increases with Geographic Distance

Total dissimilarity and dissimilarity derived from species turnover are positively correlated with geographic distance, and these relationships are more evident at the intermediate and fine grain sizes for almost all biological groups. These results reflect the phenomenon of distance-decay in similarity: similarity in species composition between sites decreases as the distance between the sites increases [47]. Three main mechanisms, which are not exclusive, have been proposed to explain this pattern [47,48]. First, environmental conditions change as the distance increases. This implies a spatial separation of species with different physiological requirements. The second mechanism depends on the configuration of the environment (both in spatial and temporal terms), because it influences the movement of species. With more barriers, similarity decreases more abruptly than in a topographically open and homogeneous site. Finally, the third mechanism is related to limitations in the dispersal ability of species, because the relationship between similarity and distance occurs even when the environment is completely homogeneous. On the Isthmus of Tehuantepec these three mechanisms may influence dissimilarity and its positive correlation with distance. However, the high environmental heterogeneity and the convergence of three physiographic subprovinces may be the most important factor. 

Despite the lack of direct evidence of the influence of dispersal ability on dissimilarity, correlations between dissimilarity and geographic distance were weaker for vagile groups (for bats the three dissimilarity measures at the three grain sizes, and for birds β_cc_ on the coarse grain grid, β_-3_, and β_rich_ at fine and intermediate grain sizes). 

Geographic distance was not related to the dissimilarity derived from richness differences for any of the analyzed grain sizes (except for amphibians at the fine grain size). This pattern was also recorded in Europe for nonflying mammals, where dissimilarity due to richness differences was not related to distance in any geographic direction (north-south, east-west), or for specific regions within the continent; species turnover was so high that even the remote cells did not represent nested subsets [45]. At small scales (less than 250 000 km^2^) high vertebrate species turnover and low richness differences results from topographic and environmental heterogeneity [44,49], while over larger scales (continents) biogeographic history plays a more important role than environmental heterogeneity does [5,7,9,10,45]. Thus, the high environmental heterogeneity of the Isthmus of Tehuantepec may influence dissimilarity due to richness differences despite the geographic distance between faunas. However, we think that this idea would benefit from an analysis carried out within each physiographic subprovince, where cells of different sizes may be nested subsets of the total fauna in the subprovince.

## Conclusions

We have shown that total dissimilarity is high for the terrestrial vertebrates, and this is explained by both dissimilarity due to richness differences and dissimilarity derived from species turnover. Also, total dissimilarity and the dissimilarity due to turnover are correlated with geographic distance, especially when we use a fine and intermediate grain size. However, we detected no clear effect of species dispersal ability on dissimilarity patterns. Our results are important to conservation biology because they indicate that the patterns of dissimilarity particular to the Isthmus of Tehuantepec must be taken into account for the protection of biodiversity. For example, besides protecting one or few extensive areas where species richness may be high, this region would benefit from a system of small, interconnected protected areas that may encompass different ecosystems and more appropriately represent most of the geographic and biological variability. Also, besides the patterns in species dissimilarity detected, efforts to protect current biodiversity should also take into account the role of historical processes, as well as the potential impact of future climate change on these patterns. 

## Supporting Information

Figure S1 **Spatial distribution of cells with more than 75% inventory completeness, according to sampling coverage.** For the geographical location of grids see Figure 1.
(PDF)

Checklist S1 **Checklist of vertebrate species in the Isthmus of Tehuantepec.**
(PDF)
