# Supplementary material for: Vertebrate Dissimilarity Due to Turnover and Richness Differences in a Highly Beta-Diverse Region: The Role of Spatial Grain Size, Dispersal Ability and Distance
Source: PLoS One. 2013 Dec 4;8(12):e82905. doi: 10.1371/journal.pone.0082905 (PMC3853624; doi:10.1371/journal.pone.0082905)

SUPPORTING INFORMATION

Vertebrate Dissimilarity due to Turnover and Richness Differences in a Highly Beta-Diverse Region: the Role of Spatial Grain Size, Dispersal Ability and Distance

Jaime M. Calderón-Patrón, Claudia E. Moreno, Rubén Pineda, Gerardo Sánchez-Rojas and Iriana Zuria

Figure S1. Spatial distribution of cells with more than 75% inventory completeness (purple cells), according to sampling coverage. For the geographical location of grids see Figure 1.

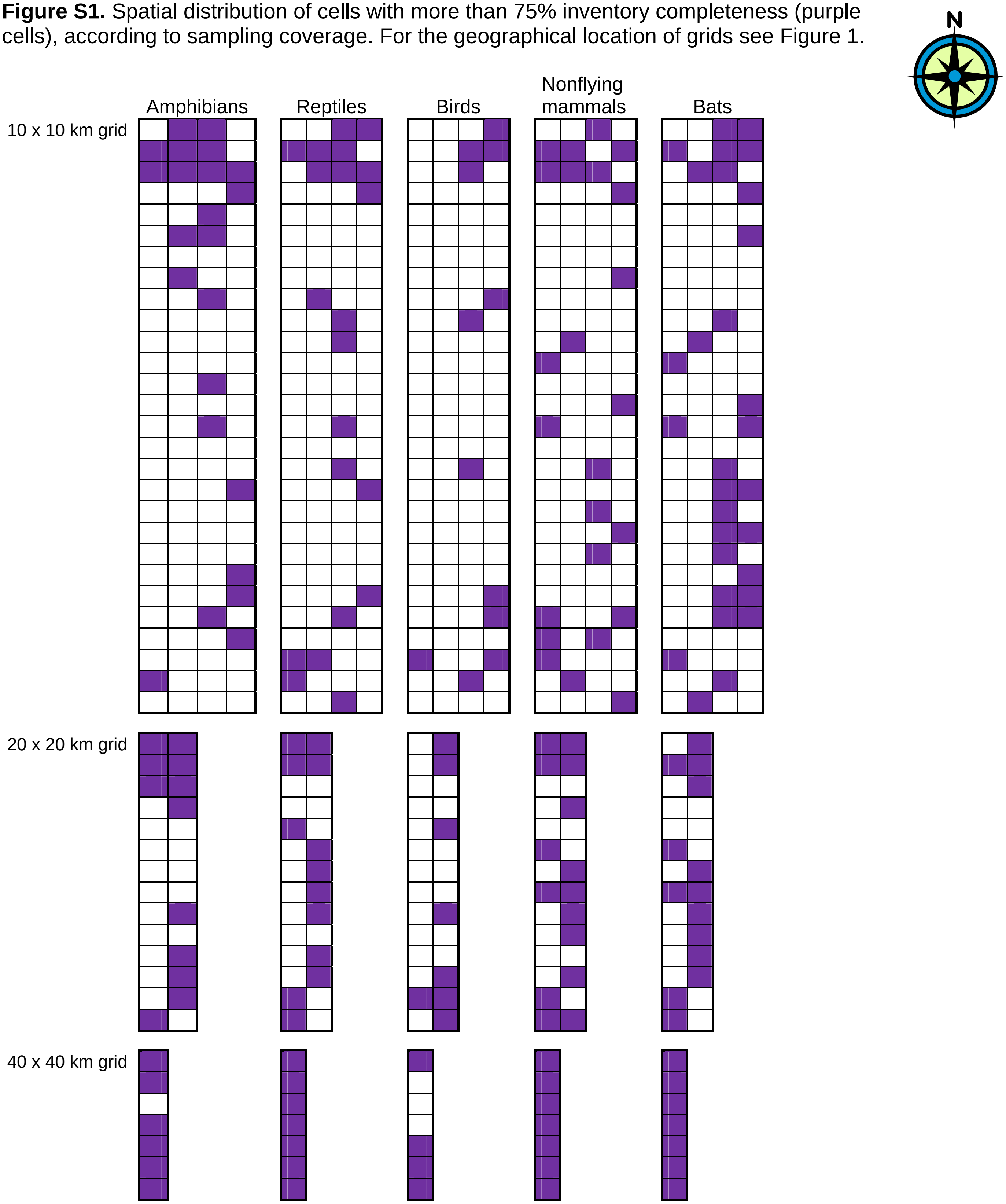

Supplement: Figure S1 — Spatial distribution of cells with more than 75% inventory completeness, according to sampling coverage. For the geographical location of grids see Figure 1. (PDF) [file pone.0082905.s001.pdf]
