# Supplementary material for: Vertebrate Dissimilarity Due to Turnover and Richness Differences in a Highly Beta-Diverse Region: The Role of Spatial Grain Size, Dispersal Ability and Distance
Source: PLoS One. 2013 Dec 4;8(12):e82905. doi: 10.1371/journal.pone.0082905 (PMC3853624; doi:10.1371/journal.pone.0082905)
Supplement: Checklist S1 — Checklist of vertebrate species in the Isthmus of Tehuantepec. (PDF) [file pone.0082905.s002.pdf]

SUPPORTING INFORMATION

Vertebrate Dissimilarity due to Turnover and Richness Differences in a Highly Beta-Diverse Region: the Role of Spatial Grain Size, Dispersal Ability and Distance

Jaime M. Calderón-Patrón, Claudia E. Moreno, Rubén Pineda, Gerardo Sánchez-Rojas and Iriana Zuria

Checklist S1. Checklist of vertebrate species in the Isthmus of Tehuantepec

Number	Group	Species
1	Amphibians	*Agalychnis callidryas*
2	Amphibians	*Anotheca spinosa*
3	Amphibians	*Bolitoglossa alberchi*
4	Amphibians	*Bolitoglossa mexicana*
5	Amphibians	*Bolitoglossa occidentalis*
6	Amphibians	*Bolitoglossa platydactyla*
7	Amphibians	*Bolitoglossa rufescens*
8	Amphibians	*Bromeliohyla dendroscarta*
9	Amphibians	*Chiropterotriton chiropterus*
10	Amphibians	*Craugastor alfredi*
11	Amphibians	*Craugastor augusti*
12	Amphibians	*Craugastor berkenbuschii*
13	Amphibians	*Craugastor laticeps*
14	Amphibians	*Craugastor loki*
15	Amphibians	*Craugastor megalotympanum*
16	Amphibians	*Craugastor mexicanus*
17	Amphibians	*Craugastor pygmaeus*
18	Amphibians	*Craugastor rhodopis*
19	Amphibians	*Craugastor rugulosus*
20	Amphibians	*Craugastor spatulatus*
21	Amphibians	*Dendropsophus ebraccatus*
22	Amphibians	*Dendropsophus microcephalus*
23	Amphibians	*Dermophis mexicanus*
24	Amphibians	*Diaglena spatulata*
25	Amphibians	*Ecnomiohyla miotympanum*
26	Amphibians	*Ecnomiohyla valancifer*
27	Amphibians	*Eleutherodactylus leprus*
28	Amphibians	*Eleutherodactylus pipilans*
29	Amphibians	*Engystomops pustulosus*
30	Amphibians	*Exerodonta sumichrasti*
31	Amphibians	*Gastrophryne elegans*
32	Amphibians	*Gastrophryne usta*
33	Amphibians	*Hyalinobatrachium fleischmanni*
34	Amphibians	*Hyla euphorbiacea*

Number	Group	Species
35	Amphibians	*Hyla eximia*
36	Amphibians	*Hypopachus variolosus*
37	Amphibians	*Incilius canaliferus*
38	Amphibians	*Incilius cavifrons*
39	Amphibians	*Incilius coccifer*
40	Amphibians	*Incilius cristatus*
41	Amphibians	*Incilius marmoreus*
42	Amphibians	*Incilius spiculatus*
43	Amphibians	*Incilius valliceps*
44	Amphibians	*Leptodactylus fragilis*
45	Amphibians	*Leptodactylus melanonotus*
46	Amphibians	*Lithobates berlandieri*
47	Amphibians	*Lithobates brownorum*
48	Amphibians	*Lithobates catesbeianus*
49	Amphibians	*Lithobates forreri*
50	Amphibians	*Lithobates maculatus*
51	Amphibians	*Lithobates pustulosus*
52	Amphibians	*Lithobates vaillanti*
53	Amphibians	*Megastomatohyla mixomaculata*
54	Amphibians	*Plectrohyla pycnochila*
55	Amphibians	*Pseudoeurycea lineola*
56	Amphibians	*Pseudoeurycea nigromaculata*
57	Amphibians	*Pseudoeurycea orchimelas*
58	Amphibians	*Pseudoeurycea werleri*
59	Amphibians	*Rhinella marina*
60	Amphibians	*Rhinophrynus dorsalis*
61	Amphibians	*Scinax staufferi*
62	Amphibians	*Smilisca baudinii*
63	Amphibians	*Smilisca cyanosticta*
64	Amphibians	*Thorius narismagnus*
65	Amphibians	*Tlalocohyla loquax*
66	Amphibians	*Tlalocohyla picta*
67	Amphibians	*Trachycephalus venulosus*
1	Reptiles	*Abronia chiszari*
2	Reptiles	*Abronia reidi*
3	Reptiles	*Abronia taeniata*
4	Reptiles	*Adelphicos latifasciatum*
5	Reptiles	*Adelphicos quadrivirgatum*
6	Reptiles	*Adelphicos visoninum*
7	Reptiles	*Agkistrodon bilineatus*

Number	Group	Species
8	Reptiles	*Amastridium sapperi*
9	Reptiles	*Ameiva undulata*
10	Reptiles	*Anolis baccatus*
11	Reptiles	*Anolis barkeri*
12	Reptiles	*Anolis biporcatus*
13	Reptiles	*Anolis compressicauda*
14	Reptiles	*Anolis cuprinus*
15	Reptiles	*Anolis duellmani*
16	Reptiles	*Anolis isthmicus*
17	Reptiles	*Anolis laeviventris*
18	Reptiles	*Anolis lemurinus*
19	Reptiles	*Anolis nebuloides*
20	Reptiles	*Anolis nebulosus*
21	Reptiles	*Anolis pentaprion*
22	Reptiles	*Anolis petersi*
23	Reptiles	*Anolis rodriguezi*
24	Reptiles	*Anolis sagrei*
25	Reptiles	*Anolis schiedeii*
26	Reptiles	*Anolis sericeus*
27	Reptiles	*Anolis tropidonotus*
28	Reptiles	*Anolis uniformis*
29	Reptiles	*Aspidoscelis costata*
30	Reptiles	*Aspidoscelis deppii*
31	Reptiles	*Aspidoscelis guttata*
32	Reptiles	*Aspidoscelis mexicana*
33	Reptiles	*Aspidoscelis motaguae*
34	Reptiles	*Aspidoscelis sacki*
35	Reptiles	*Atropoides olmec*
36	Reptiles	*Barisia imbricata*
37	Reptiles	*Basiliscus vittatus*
38	Reptiles	*Boa constrictor*
39	Reptiles	*Bothrops asper*
40	Reptiles	*Caiman crocodilus*
41	Reptiles	*Claudius angustatus*
42	Reptiles	*Clelia scytalina*
43	Reptiles	*Coleonyx elegans*
44	Reptiles	*Coluber flagellum*
45	Reptiles	*Coluber mentovarius*
46	Reptiles	*Coniophanes bipunctatus*
47	Reptiles	*Coniophanes fissidens*

Number	Group	Species
48	Reptiles	*Coniophanes imperialis*
49	Reptiles	*Coniophanes piceivittis*
50	Reptiles	*Coniophanes quinquevittatus*
51	Reptiles	*Conophis lineatus*
52	Reptiles	*Conophis vittatus*
53	Reptiles	*Conopsis acuta*
54	Reptiles	*Conopsis lineata*
55	Reptiles	*Conopsis megalodon*
56	Reptiles	*Conopsis nasus*
57	Reptiles	*Corytophanes hernandesii*
58	Reptiles	*Crocodylus acutus*
59	Reptiles	*Crocodylus moreletii*
60	Reptiles	*Crotalus atrox*
61	Reptiles	*Crotalus durissus*
62	Reptiles	*Crotalus simus*
63	Reptiles	*Ctenosaura acanthura*
64	Reptiles	*Ctenosaura clarki*
65	Reptiles	*Ctenosaura oaxacana*
66	Reptiles	*Ctenosaura pectinata*
67	Reptiles	*Ctenosaura similis*
68	Reptiles	*Dendrophidion vinitor*
69	Reptiles	*Dermatemys mawii*
70	Reptiles	*Diploglossus enneagrammus*
71	Reptiles	*Drymarchon corais*
72	Reptiles	*Drymarchon melanurus*
73	Reptiles	*Drymobius chloroticus*
74	Reptiles	*Drymobius margaritiferus*
75	Reptiles	*Enulius flavitorques*
76	Reptiles	*Eretmochelys imbricata*
77	Reptiles	*Ficimia olivaceus*
78	Reptiles	*Ficimia publia*
79	Reptiles	*Ficimia variegata*
80	Reptiles	*Geagras redimitus*
81	Reptiles	*Geophis anocularis*
82	Reptiles	*Geophis carinosus*
83	Reptiles	*Geophis dubius*
84	Reptiles	*Gerrhonotus liocephalus*
85	Reptiles	*Gymnophthalmus speciosus*
86	Reptiles	*Heloderma horridum*
87	Reptiles	*Hemidactylus frenatus*

Number	Group	Species
88	Reptiles	*Hemidactylus mabouia*
89	Reptiles	*Hemidactylus turcicus*
90	Reptiles	*Iguana iguana*
91	Reptiles	*Imantodes cenchoa*
92	Reptiles	*Imantodes gemmistratus*
93	Reptiles	*Kinosternon acutum*
94	Reptiles	*Kinosternon integrum*
95	Reptiles	*Kinosternon leucostomum*
96	Reptiles	*Kinosternon scorpioides*
97	Reptiles	*Laemanctus longipes*
98	Reptiles	*Laemanctus serratus*
99	Reptiles	*Lampropeltis triangulum*
100	Reptiles	*Lepidophyma flavimaculatum*
101	Reptiles	*Lepidophyma pajapanensis*
102	Reptiles	*Lepidophyma smithi*
103	Reptiles	*Lepidophyma tuxtlae*
104	Reptiles	*Leptodeira annulata*
105	Reptiles	*Leptodeira frenata*
106	Reptiles	*Leptodeira maculata*
107	Reptiles	*Leptodeira nigrofasciata*
108	Reptiles	*Leptodeira septentrionalis*
109	Reptiles	*Leptophis ahaetulla*
110	Reptiles	*Leptophis diplotropis*
111	Reptiles	*Leptophis mexicanus*
112	Reptiles	*Leptotyphlops goudotii*
113	Reptiles	*Loxocemus bicolor*
114	Reptiles	*Mabuya brachypoda*
115	Reptiles	*Mabuya unimarginata*
116	Reptiles	*Manolepis putnami*
117	Reptiles	*Mastigodryas melanolomus*
118	Reptiles	*Micrurus diastema*
119	Reptiles	*Micrurus elegans*
120	Reptiles	*Micrurus ephippifer*
121	Reptiles	*Micrurus latifasciatus*
122	Reptiles	*Micrurus limbatus*
123	Reptiles	*Ninia diademata*
124	Reptiles	*Ninia sebae*
125	Reptiles	*Oxybelis aeneus*
126	Reptiles	*Oxybelis fulgidus*
127	Reptiles	*Oxyrhopus petolarius*

Number	Group	Species
128	Reptiles	*Pelamis platurus*
129	Reptiles	*Phrynosoma asio*
130	Reptiles	*Phyllodactylus davisi*
131	Reptiles	*Phyllodactylus lanei*
132	Reptiles	*Phyllodactylus muralis*
133	Reptiles	*Phyllodactylus tuberculosus*
134	Reptiles	*Pituophis deppei*
135	Reptiles	*Plestiodon brevirostris*
136	Reptiles	*Plestiodon copei*
137	Reptiles	*Plestiodon sumichrasti*
138	Reptiles	*Pliocercus elapoides*
139	Reptiles	*Pliocercus wilmarai*
140	Reptiles	*Porthidium dunni*
141	Reptiles	*Pseudelaphe flavirufa*
142	Reptiles	*Pseustes poecilonotus*
143	Reptiles	*Ramphotyphlops braminus*
144	Reptiles	*Rhadinaea decorata*
145	Reptiles	*Rhadinaea fulvivittis*
146	Reptiles	*Rhadinaea taeniata*
147	Reptiles	*Rhinoclemmys areolata*
148	Reptiles	*Rhinoclemmys pulcherrima*
149	Reptiles	*Rhinoclemmys rubida*
150	Reptiles	*Salvadora bairdi*
151	Reptiles	*Salvadora lemniscata*
152	Reptiles	*Salvadora mexicana*
153	Reptiles	*Scaphiodontophis annulatus*
154	Reptiles	*Sceloporus acanthinus*
155	Reptiles	*Sceloporus consobrinus*
156	Reptiles	*Sceloporus edwardtaylori*
157	Reptiles	*Sceloporus formosus*
158	Reptiles	*Sceloporus grammicus*
159	Reptiles	*Sceloporus internasalis*
160	Reptiles	*Sceloporus melanorhinus*
161	Reptiles	*Sceloporus salvini*
162	Reptiles	*Sceloporus serrifer*
163	Reptiles	*Sceloporus siniferus*
164	Reptiles	*Sceloporus spinosus*
165	Reptiles	*Sceloporus squamosus*
166	Reptiles	*Sceloporus teapensis*
167	Reptiles	*Sceloporus variabilis*

Number	Group	Species
168	Reptiles	*Scincella gemmingeri*
169	Reptiles	*Scincella silvicola*
170	Reptiles	*Senticolis triaspis*
171	Reptiles	*Sibon dimidiatus*
172	Reptiles	*Sibon nebulatus*
173	Reptiles	*Sphaerodactylus glaucus*
174	Reptiles	*Sphaerodactylus millepunctatus*
175	Reptiles	*Sphenomorphus assatus*
176	Reptiles	*Sphenomorphus cherriei*
177	Reptiles	*Spilotes pullatus*
178	Reptiles	*Staurotypus salvinii*
179	Reptiles	*Staurotypus triporcatus*
180	Reptiles	*Stenorrhina degenhardtii*
181	Reptiles	*Stenorrhina freminvillei*
182	Reptiles	*Symphimus leucostomus*
183	Reptiles	*Tantilla briggsi*
184	Reptiles	*Tantilla deppei*
185	Reptiles	*Tantilla rubra*
186	Reptiles	*Tantilla schistosa*
187	Reptiles	*Tantilla slavensi*
188	Reptiles	*Tantilla striata*
189	Reptiles	*Tantillita lintoni*
190	Reptiles	*Thamnophis marcianus*
191	Reptiles	*Thamnophis melanogaster*
192	Reptiles	*Thamnophis proximus*
193	Reptiles	*Trachemys scripta*
194	Reptiles	*Trachemys venusta*
195	Reptiles	*Tretanorhinus nigroluteus*
196	Reptiles	*Trimorphodon biscutatus*
197	Reptiles	*Trimorphodon tau*
198	Reptiles	*Tropidodipsas fasciata*
199	Reptiles	*Tropidodipsas sartoriii*
200	Reptiles	*Typhlops tenuis*
201	Reptiles	*Urosaurus bicarinatus*
202	Reptiles	*Xenodon rabdocephalus*
203	Reptiles	*Xenosaurus grandis*
1	Birds	*Accipiter bicolor*
2	Birds	*Accipiter cooperii*
3	Birds	*Accipiter striatus*
4	Birds	*Actitis macularius*

Number	Group	Species
5	Birds	*Aeronautes saxatalis*
6	Birds	*Agelaius phoeniceus*
7	Birds	*Aimophila rufescens*
8	Birds	*Aimophila ruficauda*
9	Birds	*Aimophila sumichrasti*
10	Birds	*Amaurolimnas concolor*
11	Birds	*Amazilia beryllina*
12	Birds	*Amazilia candida*
13	Birds	*Amazilia cyanocephala*
14	Birds	*Amazilia rutila*
15	Birds	*Amazilia tzacatl*
16	Birds	*Amazilia violiceps*
17	Birds	*Amazilia viridifrons*
18	Birds	*Amazona albifrons*
19	Birds	*Amazona autumnalis*
20	Birds	*Amazona farinosa*
21	Birds	*Amazona oratrix*
22	Birds	*Amblycercus holosericeus*
23	Birds	*Ammodramus savannarum*
24	Birds	*Anas acuta*
25	Birds	*Anas americana*
26	Birds	*Anas clypeata*
27	Birds	*Anas discors*
28	Birds	*Anas platyrhynchos*
29	Birds	*Anas strepera*
30	Birds	*Anhinga anhinga*
31	Birds	*Anthracothorax prevostii*
32	Birds	*Aramides cajanea*
33	Birds	*Aramus guarauna*
34	Birds	*Aratinga canicularis*
35	Birds	*Aratinga nana*
36	Birds	*Archilochus colubris*
37	Birds	*Ardea alba*
38	Birds	*Ardea herodias*
39	Birds	*Arenaria interpres*
40	Birds	*Arremon aurantiirostris*
41	Birds	*Arremon brunneinucha*
42	Birds	*Arremonops rufivirgatus*
43	Birds	*Athene cunicularia*
44	Birds	*Attila spadiceus*

Number	Group	Species
45	Birds	*Aulacorhynchus prasinus*
46	Birds	*Automolus ochrolaemus*
47	Birds	*Aythya affinis*
48	Birds	*Bartramia longicauda*
49	Birds	*Basileuterus culicivorus*
50	Birds	*Basileuterus lachrymosus*
51	Birds	*Basileuterus rufifrons*
52	Birds	*Bombycilla cedrorum*
53	Birds	*Bubo virginianus*
54	Birds	*Bubulcus ibis*
55	Birds	*Burhinus bistriatus*
56	Birds	*Buteo albicaudatus*
57	Birds	*Buteo albonotatus*
58	Birds	*Buteo brachyurus*
59	Birds	*Buteo jamaicensis*
60	Birds	*Buteo magnirostris*
61	Birds	*Buteo nitidus*
62	Birds	*Buteo platypterus*
63	Birds	*Buteo swainsoni*
64	Birds	*Buteogallus anthracinus*
65	Birds	*Butorides virescens*
66	Birds	*Cacicus melanicterus*
67	Birds	*Cairina moschata*
68	Birds	*Calidris alba*
69	Birds	*Calidris canutus*
70	Birds	*Calidris himantopus*
71	Birds	*Calidris mauri*
72	Birds	*Calidris melanotos*
73	Birds	*Calidris minutilla*
74	Birds	*Calidris pusilla*
75	Birds	*Calocitta formosa*
76	Birds	*Calothorax pulcher*
77	Birds	*Campephilus guatemalensis*
78	Birds	*Camptostoma imberbe*
79	Birds	*Campylopterus curvipennis*
80	Birds	*Campylopterus excellens*
81	Birds	*Campylopterus hemileucurus*
82	Birds	*Campylorhynchus rufinucha*
83	Birds	*Campylorhynchus zonatus*
84	Birds	*Caprimulgus carolinensis*

Number	Group	Species
85	Birds	*Caprimulgus maculicaudus*
86	Birds	*Caprimulgus ridgwayi*
87	Birds	*Caprimulgus vociferus*
88	Birds	*Caracara cheriway*
89	Birds	*Cardellina canadensis*
90	Birds	*Cardellina pusilla*
91	Birds	*Cardinalis cardinalis*
92	Birds	*Cardinalis sinuatus*
93	Birds	*Caryothraustes poliogaster*
94	Birds	*Cathartes aura*
95	Birds	*Catharus mexicanus*
96	Birds	*Catharus ustulatus*
97	Birds	*Celeus castaneus*
98	Birds	*Chaetura pelagica*
99	Birds	*Chaetura vauxi*
100	Birds	*Charadrius collaris*
101	Birds	*Charadrius nivosus*
102	Birds	*Charadrius semipalmatus*
103	Birds	*Charadrius vociferus*
104	Birds	*Chiroxiphia linearis*
105	Birds	*Chlidonias niger*
106	Birds	*Chloroceryle aenea*
107	Birds	*Chloroceryle amazona*
108	Birds	*Chloroceryle americana*
109	Birds	*Chlorophanes spiza*
110	Birds	*Chlorostilbon auriceps*
111	Birds	*Chlorostilbon canivetii*
112	Birds	*Chondestes grammacus*
113	Birds	*Chondrohierax uncinatus*
114	Birds	*Chordeiles acutipennis*
115	Birds	*Ciccaba nigrolineata*
116	Birds	*Ciccaba virgata*
117	Birds	*Circus cyaneus*
118	Birds	*Claravis pretiosa*
119	Birds	*Coccyzus americanus*
120	Birds	*Coccyzus minor*
121	Birds	*Cochlearius cochlearius*
122	Birds	*Coereba flaveola*
123	Birds	*Colaptes rubiginosus*
124	Birds	*Colinus virginianus*

Number	Group	Species
125	Birds	*Columba livia*
126	Birds	*Columbina inca*
127	Birds	*Columbina minuta*
128	Birds	*Columbina passerina*
129	Birds	*Columbina talpacoti*
130	Birds	*Contopus cinereus*
131	Birds	*Contopus cooperi*
132	Birds	*Contopus sordidulus*
133	Birds	*Contopus virens*
134	Birds	*Coragyps atratus*
135	Birds	*Corvus corax*
136	Birds	*Cotinga amabilis*
137	Birds	*Crax rubra*
138	Birds	*Crotophaga sulcirostris*
139	Birds	*Crypturellus boucardi*
140	Birds	*Crypturellus cinnamomeus*
141	Birds	*Cyanerpes cyaneus*
142	Birds	*Cyanocompsa cyanoides*
143	Birds	*Cyanocompsa parellina*
144	Birds	*Cyanocorax yncas*
145	Birds	*Cynanthus latirostris*
146	Birds	*Cypseloides niger*
147	Birds	*Deltarhynchus flammulatus*
148	Birds	*Dendrocincla anabatina*
149	Birds	*Dendrocincla homochroa*
150	Birds	*Dendrocygna autumnalis*
151	Birds	*Dendrocygna bicolor*
152	Birds	*Diglossa baritula*
153	Birds	*Dives dives*
154	Birds	*Dolichonyx oryzivorus*
155	Birds	*Dryocopus lineatus*
156	Birds	*Dumetella carolinensis*
157	Birds	*Egretta caerulea*
158	Birds	*Egretta rufescens*
159	Birds	*Egretta thula*
160	Birds	*Egretta tricolor*
161	Birds	*Elaenia flavogaster*
162	Birds	*Elanoides forficatus*
163	Birds	*Elanus leucurus*
164	Birds	*Empidonax alnorum*

Number	Group	Species
165	Birds	*Empidonax flaviventris*
166	Birds	*Empidonax minimus*
167	Birds	*Empidonax occidentalis*
168	Birds	*Empidonax traillii*
169	Birds	*Eremophila alpestris*
170	Birds	*Eucometis penicillata*
171	Birds	*Eudocimus albus*
172	Birds	*Eumomota superciliosa*
173	Birds	*Euphagus cyanocephalus*
174	Birds	*Eupherusa eximia*
175	Birds	*Euphonia affinis*
176	Birds	*Euphonia elegantissima*
177	Birds	*Euphonia gouldi*
178	Birds	*Euphonia hirundinacea*
179	Birds	*Falco columbarius*
180	Birds	*Falco femoralis*
181	Birds	*Falco peregrinus*
182	Birds	*Falco rufigularis*
183	Birds	*Falco sparverius*
184	Birds	*Formicarius analis*
185	Birds	*Fregata magnificens*
186	Birds	*Fulica americana*
187	Birds	*Galbula ruficauda*
188	Birds	*Gallinula galeata*
189	Birds	*Gelochelidon nilotica*
190	Birds	*Geococcyx velox*
191	Birds	*Geothlypis formosa*
192	Birds	*Geothlypis philadelphia*
193	Birds	*Geothlypis poliocephala*
194	Birds	*Geothlypis tolmiei*
195	Birds	*Geothlypis trichas*
196	Birds	*Geotrygon carrikeri*
197	Birds	*Geotrygon montana*
198	Birds	*Geranospiza caerulescens*
199	Birds	*Glaucidium brasilianum*
200	Birds	*Glyphorynchus spirurus*
201	Birds	*Habia fuscicauda*
202	Birds	*Habia rubica*
203	Birds	*Haematopus palliatus*
204	Birds	*Haemorhous mexicanus*

Number	Group	Species
205	Birds	*Harpagus bidentatus*
206	Birds	*Heliomaster constantii*
207	Birds	*Heliomaster longirostris*
208	Birds	*Helmitheros vermivorum*
209	Birds	*Henicorhina leucosticta*
210	Birds	*Herpetotheres cachinnans*
211	Birds	*Himantopus mexicanus*
212	Birds	*Hirundo rustica*
213	Birds	*Hydroprogne caspia*
214	Birds	*Hylocichla mustelina*
215	Birds	*Hylomanes momotula*
216	Birds	*Hylophilus decurtatus*
217	Birds	*Hylophilus ochraceiceps*
218	Birds	*Icteria virens*
219	Birds	*Icterus cucullatus*
220	Birds	*Icterus dominicensis*
221	Birds	*Icterus galbula*
222	Birds	*Icterus gularis*
223	Birds	*Icterus mesomelas*
224	Birds	*Icterus pectoralis*
225	Birds	*Icterus pustulatus*
226	Birds	*Icterus spurius*
227	Birds	*Ictinia mississippiensis*
228	Birds	*Ixobrychus exilis*
229	Birds	*Jacana spinosa*
230	Birds	*Lanio aurantius*
231	Birds	*Lanius ludovicianus*
232	Birds	*Laterallus ruber*
233	Birds	*Legatus leucophaius*
234	Birds	*Lepidocolaptes leucogaster*
235	Birds	*Lepidocolaptes souleyetii*
236	Birds	*Leptodon cayanensis*
237	Birds	*Leptopogon amaurocephalus*
238	Birds	*Leptotila plumbeiceps*
239	Birds	*Leptotila verreauxi*
240	Birds	*Leucophaeus atricilla*
241	Birds	*Leucophaeus pipixcan*
242	Birds	*Leucopternis albicollis*
243	Birds	*Limnodromus griseus*
244	Birds	*Limosa fedoa*

Number	Group	Species
245	Birds	*Lophornis helenae*
246	Birds	*Lophostrix cristata*
247	Birds	*Manacus candei*
248	Birds	*Megaceryle torquata*
249	Birds	*Megarynchus pitangua*
250	Birds	*Megascops cooperi*
251	Birds	*Melanerpes aurifrons*
252	Birds	*Melanerpes chrysogenys*
253	Birds	*Melanerpes formicivorus*
254	Birds	*Melanerpes pucherani*
255	Birds	*Melospiza lincolnii*
256	Birds	*Micrastur ruficollis*
257	Birds	*Micrastur semitorquatus*
258	Birds	*Mimus gilvus*
259	Birds	*Mimus polyglottos*
260	Birds	*Mionectes oleagineus*
261	Birds	*Mniotilta varia*
262	Birds	*Molothrus aeneus*
263	Birds	*Molothrus ater*
264	Birds	*Momotus mexicanus*
265	Birds	*Momotus momota*
266	Birds	*Morococcyx erythropygus*
267	Birds	*Myadestes unicolor*
268	Birds	*Mycteria americana*
269	Birds	*Myiarchus cinerascens*
270	Birds	*Myiarchus crinitus*
271	Birds	*Myiarchus nuttingi*
272	Birds	*Myiarchus tuberculifer*
273	Birds	*Myiarchus tyrannulus*
274	Birds	*Myiobius sulphureipygius*
275	Birds	*Myiodynastes luteiventris*
276	Birds	*Myiodynastes maculatus*
277	Birds	*Myiopagis viridicata*
278	Birds	*Myiozetetes similis*
279	Birds	*Numenius americanus*
280	Birds	*Numenius phaeopus*
281	Birds	*Nyctanassa violacea*
282	Birds	*Nyctibius jamaicensis*
283	Birds	*Nycticorax nycticorax*
284	Birds	*Nyctidromus albicollis*

Number	Group	Species
285	Birds	*Odontophorus guttatus*
286	Birds	*Oncostoma cinereigulare*
287	Birds	*Oreothlypis peregrina*
288	Birds	*Oreothlypis ruficapilla*
289	Birds	*Ornithion semiflavum*
290	Birds	*Ortalis leucogastra*
291	Birds	*Ortalis poliocephala*
292	Birds	*Ortalis vetula*
293	Birds	*Pachyramphus aglaiae*
294	Birds	*Pandion haliaetus*
295	Birds	*Parabuteo unicinctus*
296	Birds	*Parkesia motacilla*
297	Birds	*Parkesia noveboracensis*
298	Birds	*Passer domesticus*
299	Birds	*Passerina caerulea*
300	Birds	*Passerina ciris*
301	Birds	*Passerina cyanea*
302	Birds	*Passerina leclancherii*
303	Birds	*Passerina rositae*
304	Birds	*Passerina versicolor*
305	Birds	*Patagioenas cayennensis*
306	Birds	*Patagioenas flavirostris*
307	Birds	*Patagioenas nigrirostris*
308	Birds	*Pelecanus erythrorhynchos*
309	Birds	*Pelecanus occidentalis*
310	Birds	*Penelopina nigra*
311	Birds	*Petrochelidon pyrrhonota*
312	Birds	*Peucaea ruficauda*
313	Birds	*Peucaea sumichrasti*
314	Birds	*Phaethornis longirostris*
315	Birds	*Phaethornis striigularis*
316	Birds	*Phalacrocorax brasilianus*
317	Birds	*Phalaropus tricolor*
318	Birds	*Pheucticus ludovicianus*
319	Birds	*Piaya cayana*
320	Birds	*Picoides scalaris*
321	Birds	*Pipilo erythrophthalmus*
322	Birds	*Pipra mentalis*
323	Birds	*Piranga flava*
324	Birds	*Piranga leucoptera*

Number	Group	Species
325	Birds	*Piranga ludoviciana*
326	Birds	*Piranga rubra*
327	Birds	*Pitangus sulphuratus*
328	Birds	*Platalea ajaja*
329	Birds	*Platyrinchus cancrominus*
330	Birds	*Pluvialis squatarola*
331	Birds	*Podiceps nigricollis*
332	Birds	*Podilymbus podiceps*
333	Birds	*Polioptila albiloris*
334	Birds	*Polioptila caerulea*
335	Birds	*Porphyrio martinica*
336	Birds	*Porzana carolina*
337	Birds	*Porzana flaviventer*
338	Birds	*Progne chalybea*
339	Birds	*Progne subis*
340	Birds	*Protonotaria citrea*
341	Birds	*Psarocolius montezuma*
342	Birds	*Psarocolius wagleri*
343	Birds	*Pseudoscops clamator*
344	Birds	*Psilorhinus morio*
345	Birds	*Pteroglossus torquatus*
346	Birds	*Ptilogonys cinereus*
347	Birds	*Pulsatrix perspicillata*
348	Birds	*Pyrilia haematotis*
349	Birds	*Pyrocephalus rubinus*
350	Birds	*Quiscalus mexicanus*
351	Birds	*Ramphastos sulfuratus*
352	Birds	*Ramphocaenus melanurus*
353	Birds	*Ramphocelus passerinii*
354	Birds	*Ramphocelus sanguinolentus*
355	Birds	*Recurvirostra americana*
356	Birds	*Rhynchocyclus brevirostris*
357	Birds	*Riparia riparia*
358	Birds	*Rostrhamus sociabilis*
359	Birds	*Rynchops niger*
360	Birds	*Saltator atriceps*
361	Birds	*Saltator coerulescens*
362	Birds	*Saltator maximus*
363	Birds	*Sarcoramphus papa*
364	Birds	*Sayornis nigricans*

Number	Group	Species
365	Birds	*Sayornis phoebe*
366	Birds	*Scaphidura oryzivora*
367	Birds	*Schiffornis turdina*
368	Birds	*Sclerurus mexicanus*
369	Birds	*Seiurus aurocapilla*
370	Birds	*Setophaga americana*
371	Birds	*Setophaga cerulea*
372	Birds	*Setophaga citrina*
373	Birds	*Setophaga coronata*
374	Birds	*Setophaga dominica*
375	Birds	*Setophaga fusca*
376	Birds	*Setophaga magnolia*
377	Birds	*Setophaga palmarum*
378	Birds	*Setophaga pensylvanica*
379	Birds	*Setophaga petechia*
380	Birds	*Setophaga pinus*
381	Birds	*Setophaga ruticilla*
382	Birds	*Setophaga tigrina*
383	Birds	*Setophaga virens*
384	Birds	*Sittasomus griseicapillus*
385	Birds	*Spiza americana*
386	Birds	*Spizaetus ornatus*
387	Birds	*Spizaetus tyrannus*
388	Birds	*Sporophila americana*
389	Birds	*Sporophila minuta*
390	Birds	*Sporophila torqueola*
391	Birds	*Stelgidopteryx serripennis*
392	Birds	*Sterna forsteri*
393	Birds	*Sternula antillarum*
394	Birds	*Streptoprocne zonaris*
395	Birds	*Strix varia*
396	Birds	*Sturnella magna*
397	Birds	*Sula sula*
398	Birds	*Synallaxis erythrothorax*
399	Birds	*Tachybaptus dominicus*
400	Birds	*Tachycineta albilinea*
401	Birds	*Tachycineta thalassina*
402	Birds	*Taraba major*
403	Birds	*Thalasseus maximus*
404	Birds	*Thalasseus sandvicensis*

Number	Group	Species
405	Birds	*Thamnophilus doliatus*
406	Birds	*Thraupis abbas*
407	Birds	*Thraupis episcopus*
408	Birds	*Thryothorus maculipectus*
409	Birds	*Thryothorus pleurostictus*
410	Birds	*Tiaris olivaceus*
411	Birds	*Tigrisoma lineatum*
412	Birds	*Tigrisoma mexicanum*
413	Birds	*Tinamus major*
414	Birds	*Tityra inquisitor*
415	Birds	*Tityra semifasciata*
416	Birds	*Todirostrum cinereum*
417	Birds	*Tolmomyias sulphurescens*
418	Birds	*Toxostoma curvirostre*
419	Birds	*Tringa flavipes*
420	Birds	*Tringa incana*
421	Birds	*Tringa melanoleuca*
422	Birds	*Tringa semipalmata*
423	Birds	*Tringa solitaria*
424	Birds	*Troglodytes aedon*
425	Birds	*Trogon caligatus*
426	Birds	*Trogon citreolus*
427	Birds	*Trogon collaris*
428	Birds	*Trogon massena*
429	Birds	*Trogon melanocephalus*
430	Birds	*Turdus assimilis*
431	Birds	*Turdus grayi*
432	Birds	*Turdus rufopalliatus*
433	Birds	*Tyrannus couchii*
434	Birds	*Tyrannus crassirostris*
435	Birds	*Tyrannus forficatus*
436	Birds	*Tyrannus melancholicus*
437	Birds	*Tyrannus savana*
438	Birds	*Tyrannus tyrannus*
439	Birds	*Tyrannus verticalis*
440	Birds	*Tyto alba*
441	Birds	*Uropsila leucogastra*
442	Birds	*Veniliornis fumigatus*
443	Birds	*Vermivora chrysoptera*
444	Birds	*Vermivora cyanoptera*

Number	Group	Species
445	Birds	*Vireo bellii*
446	Birds	*Vireo flavifrons*
447	Birds	*Vireo flavoviridis*
448	Birds	*Vireo gilvus*
449	Birds	*Vireo griseus*
450	Birds	*Vireo olivaceus*
451	Birds	*Vireo pallens*
452	Birds	*Vireo philadelphicus*
453	Birds	*Vireo solitarius*
454	Birds	*Volatinia jacarina*
455	Birds	*Xenops minutus*
456	Birds	*Xiphorhynchus flavigaster*
457	Birds	*Zenaida asiatica*
458	Birds	*Zenaida macroura*
1	Nonflying mammals	*Alouatta palliata*
2	Nonflying mammals	*Ateles geoffroyi*
3	Nonflying mammals	*Baiomys musculus*
4	Nonflying mammals	*Bassariscus astutus*
5	Nonflying mammals	*Bassariscus sumichrasti*
6	Nonflying mammals	*Caluromys derbianus*
7	Nonflying mammals	*Canis latrans*
8	Nonflying mammals	*Chironectes minimus*
9	Nonflying mammals	*Coendou mexicanus*
10	Nonflying mammals	*Conepatus leuconotus*
11	Nonflying mammals	*Conepatus semistriatus*
12	Nonflying mammals	*Cratogeomys merriami*
13	Nonflying mammals	*Cryptotis goldmani*
14	Nonflying mammals	*Cryptotis magna*
15	Nonflying mammals	*Cryptotis mexicana*
16	Nonflying mammals	*Cryptotis parva*
17	Nonflying mammals	*Cuniculus paca*
18	Nonflying mammals	*Cyclopes didactylus*
19	Nonflying mammals	*Dasyprocta mexicana*
20	Nonflying mammals	*Dasypus novemcinctus*
21	Nonflying mammals	*Didelphis marsupialis*
22	Nonflying mammals	*Didelphis virginiana*
23	Nonflying mammals	*Eira barbara*
24	Nonflying mammals	*Galictis vittata*
25	Nonflying mammals	*Herpailurus yagouaroundi*
26	Nonflying mammals	*Heteromys desmarestianus*

Number	Group	Species
27	Nonflying mammals	*Leopardus pardalis*
28	Nonflying mammals	*Leopardus wiedii*
29	Nonflying mammals	*Lepus callotis*
30	Nonflying mammals	*Lepus flavigularis*
31	Nonflying mammals	*Liomys irroratus*
32	Nonflying mammals	*Liomys pictus*
33	Nonflying mammals	*Lontra longicaudis*
34	Nonflying mammals	*Lynx rufus*
35	Nonflying mammals	*Marmosa mexicana*
36	Nonflying mammals	*Mazama americana*
37	Nonflying mammals	*Mephitis macroura*
38	Nonflying mammals	*Microtus quasiater*
39	Nonflying mammals	*Mustela frenata*
40	Nonflying mammals	*Nasua narica*
41	Nonflying mammals	*Neotoma mexicana*
42	Nonflying mammals	*Nyctomys sumichrasti*
43	Nonflying mammals	*Odocoileus virginianus*
44	Nonflying mammals	*Oligoryzomys fulvescens*
45	Nonflying mammals	*Orthogeomys grandis*
46	Nonflying mammals	*Orthogeomys hispidus*
47	Nonflying mammals	*Oryzomys alfaroi*
48	Nonflying mammals	*Oryzomys chapmani*
49	Nonflying mammals	*Oryzomys couesi*
50	Nonflying mammals	*Oryzomys melanotis*
51	Nonflying mammals	*Oryzomys rostratus*
52	Nonflying mammals	*Panthera onca*
53	Nonflying mammals	*Pecari tajacu*
54	Nonflying mammals	*Peromyscus aztecus*
55	Nonflying mammals	*Peromyscus beatae*
56	Nonflying mammals	*Peromyscus boylii*
57	Nonflying mammals	*Peromyscus furvus*
58	Nonflying mammals	*Peromyscus leucopus*
59	Nonflying mammals	*Peromyscus levipes*
60	Nonflying mammals	*Peromyscus megalops*
61	Nonflying mammals	*Peromyscus melanocarpus*
62	Nonflying mammals	*Peromyscus melanophrys*
63	Nonflying mammals	*Peromyscus melanurus*
64	Nonflying mammals	*Peromyscus mexicanus*
65	Nonflying mammals	*Philander opossum*
66	Nonflying mammals	*Potos flavus*

Number	Group	Species
67	Nonflying mammals	*Procyon lotor*
68	Nonflying mammals	*Puma concolor*
69	Nonflying mammals	*Reithrodontomys fulvescens*
70	Nonflying mammals	*Reithrodontomys gracilis*
71	Nonflying mammals	*Reithrodontomys megalotis*
72	Nonflying mammals	*Reithrodontomys mexicanus*
73	Nonflying mammals	*Reithrodontomys sumichrasti*
74	Nonflying mammals	*Sciurus deppei*
75	Nonflying mammals	*Sigmodon alleni*
76	Nonflying mammals	*Sigmodon hispidus*
77	Nonflying mammals	*Sigmodon mascotensis*
78	Nonflying mammals	*Spermophilus perotensis*
79	Nonflying mammals	*Spilogale gracilis*
80	Nonflying mammals	*Spilogale pygmaea*
81	Nonflying mammals	*Sylvilagus brasiliensis*
82	Nonflying mammals	*Sylvilagus cunicularius*
83	Nonflying mammals	*Sylvilagus floridanus*
84	Nonflying mammals	*Tamandua mexicana*
85	Nonflying mammals	*Tapirus bairdii*
86	Nonflying mammals	*Tayassu pecari*
87	Nonflying mammals	*Tlacuatzin canescens*
88	Nonflying mammals	*Tylomys nudicaudus*
89	Nonflying mammals	*Urocyon cinereoargenteus*
1	Bats	*Anoura geoffroyi*
2	Bats	*Artibeus aztecus*
3	Bats	*Artibeus jamaicensis*
4	Bats	*Artibeus lituratus*
5	Bats	*Artibeus phaeotis*
6	Bats	*Artibeus toltecus*
7	Bats	*Artibeus watsoni*
8	Bats	*Balantiopteryx io*
9	Bats	*Balantiopteryx plicata*
10	Bats	*Bauerus dubiaquercus*
11	Bats	*Carollia perspicillata*
12	Bats	*Carollia sowelli*
13	Bats	*Carollia subrufa*
14	Bats	*Centronycteris centralis*
15	Bats	*Centurio senex*
16	Bats	*Chiroderma salvini*
17	Bats	*Chiroderma villosum*

Number	Group	Species
18	Bats	*Choeroniscus godmani*
19	Bats	*Chrotopterus auritus*
20	Bats	*Corynorhinus mexicanus*
21	Bats	*Corynorhinus townsendii*
22	Bats	*Cynomops mexicanus*
23	Bats	*Desmodus rotundus*
24	Bats	*Diclidurus albus*
25	Bats	*Diphylla ecaudata*
26	Bats	*Enchisthenes hartii*
27	Bats	*Eptesicus brasiliensis*
28	Bats	*Eptesicus furinalis*
29	Bats	*Eptesicus fuscus*
30	Bats	*Eumops glaucinus*
31	Bats	*Eumops underwoodi*
32	Bats	*Glossophaga commissarisi*
33	Bats	*Glossophaga leachii*
34	Bats	*Glossophaga morenoi*
35	Bats	*Glossophaga soricina*
36	Bats	*Hylonycteris underwoodi*
37	Bats	*Lampronycteris brachyotis*
38	Bats	*Lasiurus blossevillii*
39	Bats	*Lasiurus cinereus*
40	Bats	*Lasiurus ega*
41	Bats	*Lasiurus intermedius*
42	Bats	*Leptonycteris nivalis*
43	Bats	*Leptonycteris yerbabuenae*
44	Bats	*Lonchorhina aurita*
45	Bats	*Lophostoma brasiliense*
46	Bats	*Macrotus waterhousii*
47	Bats	*Micronycteris microtis*
48	Bats	*Mimon cozumelae*
49	Bats	*Molossus aztecus*
50	Bats	*Molossus molossus*
51	Bats	*Molossus pretiosus*
52	Bats	*Molossus rufus*
53	Bats	*Mormoops megalophylla*
54	Bats	*Myotis elegans*
55	Bats	*Myotis fortidens*
56	Bats	*Myotis keaysi*
57	Bats	*Myotis nigricans*

Number	Group	Species
58	Bats	*Myotis velifer*
59	Bats	*Natalus stramineus*
60	Bats	*Noctilio leporinus*
61	Bats	*Nyctinomops femorosaccus*
62	Bats	*Nyctinomops laticaudatus*
63	Bats	*Perimyotis subflavus*
64	Bats	*Peropteryx kappleri*
65	Bats	*Peropteryx macrotis*
66	Bats	*Phyllostomus discolor*
67	Bats	*Platyrrhinus helleri*
68	Bats	*Promops centralis*
69	Bats	*Pteronotus davyi*
70	Bats	*Pteronotus gymnonotus*
71	Bats	*Pteronotus parnellii*
72	Bats	*Pteronotus personatus*
73	Bats	*Rhogeessa gracilis*
74	Bats	*Rhogeessa parvula*
75	Bats	*Rhogeessa tumida*
76	Bats	*Rhynchonycteris naso*
77	Bats	*Saccopteryx bilineata*
78	Bats	*Sturnira lilium*
79	Bats	*Sturnira ludovici*
80	Bats	*Tadarida brasiliensis*
81	Bats	*Thyroptera tricolor*
82	Bats	*Trachops cirrhosus*
83	Bats	*Uroderma bilobatum*
84	Bats	*Uroderma magnirostrum*
85	Bats	*Vampyressa thyone*
86	Bats	*Vampyrodes caraccioli*
87	Bats	*Vampyrum spectrum*
